# Comparison of acid-detergent lignin, alkaline-peroxide lignin, and acid-detergent insoluble ash as internal markers for predicting fecal output and digestibility by cattle offered bermudagrass hays of varying nutrient composition

**DOI:** 10.1186/2049-1891-5-7

**Published:** 2014-01-13

**Authors:** Juvenal Kanani, Dirk Philipp, Kenneth P Coffey, Elizabeth B Kegley, Charles P West, Shane Gadberry, John Jennings, Ashley N Young, Robert T Rhein

**Affiliations:** 1 Division of Agriculture, University of Arkansas, Fayetteville, AR 72701, USA; 2 Department of Plant and Soil Science, Texas Tech University, Lubbock, TX 79409, USA

**Keywords:** Acid-detergent insoluble ash, Alkaline-peroxide lignin, Bermudagrass, Cattle, Digestibility, Marker

## Abstract

**Background:**

The potential for acid-detergent insoluble ash (ADIA), alkaline-peroxide lignin (APL), and acid-detergent lignin (ADL) to predict fecal output (FO) and dry matter digestibility (DMD) by cattle offered bermudagrass [*Cynodon dactylon* (L.) Pers.] hays of different qualities was evaluated. Eight ruminally cannulated cows (594 ± 35.5 kg) were allocated randomly to 4 hay diets: low (L), medium low (ML), medium high (MH), and high (H) crude protein (CP) concentration (79, 111, 131, and 164 g CP/kg on a DM basis, respectively). Diets were offered in 3 periods with 2 diet replicates per period and were rotated across cows between periods. Cows were individually fed 20 g DM/kg of body weight in equal feedings at 08:00 and 16:00 h for a 10-d adaptation followed by a 5-d total fecal collection. Actual DM intake (DMI), DMD, and FO were determined based on hay offered, ort, and feces excreted. These components were then analyzed for ADL, APL, and ADIA concentration to determine marker recovery and marker-based estimates of FO and DMD.

**Results:**

Forage DMI was affected by diet (*P =* 0.02), and DMI from MH and H was greater (*P* < 0.05) than from L. Apparent DMD tended (*P* = 0.08) to differ among diets while FO (*P =* 0.20) was not affected by diet treatments. Average ADL recovery (1.16) was greater (*P <* 0.05) than that of ADIA (1.03) and APL (1.06), but ADIA and APL did not differ (*P =* 0.42). Estimates of FO and DMD derived using APL and ADIA were not different (*P* ≥ 0.05) from total fecal collection while those using ADL differed (*P <* 0.05). There was no diet by marker interaction (*P* ≥ 0.22) for either FO or DMD.

**Conclusion:**

Acid-detergent insoluble ash and APL accurately predicted FO and DMD of cattle fed bermudagrass hay of varying nutrient composition. These internal markers may facilitate studies involving large numbers of animals and forages. Results from such studies may be used to develop improved equations to predict energy values of forages based on the relationship of dietary components to digestibility across a wide range of forages.

## Background

Traditionally, dry matter digestibility (DMD) has been determined through the *in vivo* total fecal collection procedure. Although considered the most accurate, this procedure is labor-intensive and time-consuming for evaluating a wide range of feed samples requiring a large number of animals. In an attempt to overcome these problems, indirect methods using internal markers have been proposed [1-3] which require the determination of the concentration of the markers and any other nutrient of interest in representative samples of diet and feces. The use of internal markers to estimate DMD is possible under the assumption that they are completely recoverable in feces.

Although lignin has been considered to be indigestible and recoverable in feces for many yr [4-6], recent studies indicated that lignin may not be an adequate internal marker because of potential degradability or formation of insoluble carbohydrate complexes during its transit in the gastrointestinal tract of ruminants [1,7]. However, the addition of alkaline hydrogen peroxide solution before acid-detergent fiber (ADF) analysis appeared to improve the recoveries of lignin from plants and feces [8]. Digestibility estimates using alkaline-peroxide lignin (APL) in that trial were similar to those of total fecal collection estimates when sheep were fed either immature or dormant grasses. In another digestion trial using lambs, APL gave variable digestibility estimates, even though lignin recovery was estimated to be near 100% [9]. In comparison, acid-detergent insoluble ash (ADIA) has been presented as a reliable internal marker [10], but this marker may be susceptible to soil contamination during the feeding process [11].

The variability of internal markers in predicting digestibility across different types of forages [12] requires a validation of marker recovery on a specific diet before its application in research. Little is known about internal marker recovery across different qualities of bermudagrass, which constitutes an important warm-season perennial grass for beef farms in the southern US. Therefore, the objective of this study was to evaluate ADL, APL and ADIA to be used as internal markers to determine FO and DMD of bermudagrass hay of various qualities by cattle.

## Materials and methods

The protocol used in this research was approved by the Institutional Animal Care and Use Committee of the University of Arkansas (IACUC approved protocol #10016).

### Location, treatments, and experimental design

The study was conducted at the University of Arkansas Division of Agriculture Watershed Research and Education Center (WREC) located in Fayetteville, AR. Eight ruminally cannulated dry cows (594 ± 35.5 kg) were stratified by weight and allocated to 1 of 2 blocks. Each block of 4 cows was assigned to a 4 × 3 Youden square design [13]. Four diet treatments of bermudagrass hay were duplicated in the 2 squares. The 4 bermudagrass hays varied in nutritional quality and were designated based on their crude protein (CP) concentrations: low (L, CP = 79 ± 6.2 g/kg DM); medium low (ML, CP = 111 ± 3.8 g/kg DM); medium high (MH, CP = 131 ± 4.3 g/kg DM); and high (H, CP = 164 ± 9.5 g/kg DM; see Table 1 for chemical composition of diet treatments). The combination of 8 cows used for 3 periods resulted in 24 total *in vivo* observations or 6 observations per hay treatment. Each period consisted of a 10-d adaptation period followed by 5 d of total fecal collection.

**Table 1:** **Chemical composition (g/kg dry matter, DM) of bermudagrass hays fed during an *in vivo *** **experiment for estimating marker recovery based on different crude protein (CP) levels (n = 3)**

**Item**	**Treatments**^**1**^				**SEM**^**2**^	***P*-value**
	**L**	**ML**	**MH**	**H**		
DM	885	872	867	875	10.6	0.75
Organic matter	939^a^	913^b^	912^b^	919^b^	8.2	0.04
Total ash	61	87	88	81	6.0	0.05
CP	79^c^	111^b^	131^b^	164^a^	6.4	0.01
Neutral-detergent fiber	768	712	690	740	19.1	0.08
Acid-detergent fiber	428^a^	348^b^	332^b^	370^ab^	19.4	0.03
Hemicellulose	340	364	358	370	9.1	0.19
Acid-detergent lignin	45^a^	33^b^	31^b^	41^ab^	2.9	0.03

^1^L, low CP hay (CP = 79 g/kg DM); ML, medium low CP hay (CP = 111 g/kg DM); MH, medium high CP hay (CP = 131 g/kg DM); and H, high CP hay (CP = 164 g/kg DM).

^2^SEM, standard error of the mean.

^abc^Means with different superscripts in a row differ at *P* < 0.05.

Cows were housed individually in 3.0 m × 4.3 m pens with solid concrete floors covered with rubber mats. Cows were allowed to move freely within their respective pens but were tethered during the collection period. Each pen was fitted with plastic sheets on the rails between the pens to avoid cross-contamination of feces. Cows were moved from their pens and allowed to graze and exercise for 14 d between each period to reduce the carryover effects of the previous hay treatment.

### Hay acquisition

Bermudagrass hay used in this study was selected from 3 different locations (University of Arkansas Livestock and Forestry Research and Extension Station near Batesville, AR, Animal Science departmental farm at Fayetteville, AR, and University of Arkansas Southeast Research and Extension Center in Monticello, AR) to represent a range in quality and maturity to test how well the different markers would predict the digestibility of bermudagrass hays having a range in quality. Twelve round bales were selected that weighed between 364 to 500 kg and had an average bale dimension of 1.2 m × 1.5 m. Core samples from each bale (n = 3) were taken with a Star Quality Sampler (Edmond, AB, Canada) at the round side at three different locations to a depth of 0.46 m. Samples were analyzed for CP, and based on CP, bales were allocated, irrespective of source, into the 4 hay CP groups described previously. One bale from each treatment (total of 12) was fed to 2 cows during each period. A total of 12 round bales were used for the 45-d feeding of the 3 periods.

### Feeding and sample collection

Hay was offered at a total of 20 g/kg of body weight (as-fed basis) as long hay in equal amounts at 08:00 and 16:00. The ration was formulated to meet the energy and protein requirement for a 594-kg beef cow dry consuming bermudagrass hay with an average CP of 121 g/kg DM and average total digestible nutrients of 590 g/kg DM. This restricted feeding level was chosen to minimize refusal. Water was provided ad libitum via rubber water tanks, and each cow received 114 g of a commercial cattle mineral-vitamin supplement (Purina Wind and Rain® All Season 7.5 Complete, Land O’Lakes Purina Feed LLC, Montgomery, MO, USA, containing 135 to 160 g/kg Ca, 75 g/kg P, 182.5 to 217.5 g/kg salt, and not less than 5 g/kg Mg, 10 g/kg K, 3.6 g/kg Zn, 2.115 g/kg Mn, 1.1 g/kg Cu, 0.050 g/kg Co, 0.115 g/kg I, 0.027 g/kg Se, 66,000 IU/kg Vitamin A, 66000 IU/kg Vitamin D, and 660 IU/kg Vitamin E) per day every morning before feeding hay. Feed sampling began on d 9, orts on d 10, and feces on d 11 [14]. Samples of each hay offered were taken at each feeding sequence, placed in paper bags, weighed immediately, and dried in a forced-air oven at 50°C until no further weight loss was detected. Orts (refusals) were collected each morning before feeding (07:00), weighed, and a representative sample was placed in a paper bag, weighed, and dried in a forced-air oven at 50°C until no further weight loss was detected. Total feces from each cow were collected throughout the day beginning at 08:00 on d 11 by scraping feces directly from the rubber mats. Feces were stored temporarily in plastic-lined trash cans. At 08:00 each day, total feces per cow were weighed, mixed in a commercial concrete mixer (Mixer Model 043206 Type A, Monarch Industries Inc., Canada), and a representative fecal sample (approximately 300 g of fresh feces) from the individual total daily fecal excretion was taken. This sample was then placed on paper or aluminum plates and dried to a constant weight in a forced-air oven at 50°C for determination of total FO and subsequent analysis of marker concentrations.

### Chemical analysis of ADL, APL, and ADIA

Forage, ort, and fecal samples collected during the *in vivo* experiment were ground to pass a 1-mm screen using a Wiley mill (Arthur H. Thomas Scientific, Philadelphia, PA, USA). Forage chemical compositions were analyzed for DM, total ash (TA), and total N by AOAC method 2001.12 and 2001.11 [15], respectively. Organic matter was calculated as the weight lost from combustion of DM. Neutral-detergent fiber and ADF in forage were analyzed sequentially by the batch procedure outlined by ANKOM Technology Corp. (Fairport, NY, USA). Sodium sulfite or heat-stable α-amylase was not added to the neutral-detergent solution. Hemicellulose concentrations were obtained as the difference between NDF and ADF. Acid-detergent lignin concentrations in forage, ort, and feces were analyzed using the same procedure with the omission of NDF extraction step.

#### ADL procedure

Acid-detergent lignin in ort and feces was analyzed according to the batch procedures outlined by ANKOM Technology Corp. (Fairport, NY, USA). Samples were run in duplicate. In case the coefficient of variation was greater than 5%, samples were rerun until the coefficient of variation was equal to or less than 5%.

#### APL procedure

To overcome the problem of inconsistencies in lignin recovery, the ADL procedure was modified to include an alkaline hydrogen peroxide pretreatment of samples before the ADF analysis [8]. Alkaline-peroxide lignin was isolated by pre-treating forage, ort, and fecal samples in alkaline hydrogen peroxide solution (1% H_2_O_2_ + NaOH) with pH adjusted to 11.5. This procedure was an updated combination of batch procedures (ANKOM Technology Corp., Fairport, NY, USA) and other procedures [8,12] for fiber analysis. Samples of forage, ort, and feces (0.5 ± 0.01 g) were placed directly into filter bags (ANKOM Technology Corp. #F57, Fairport, NY, USA). The bags were sealed, 24 bags each were placed into 2,000 mL beakers, then alkaline hydrogen peroxide solution was added at a rate of 50 mL alkaline hydrogen peroxide solution per bag. The bags were incubated for 24 h with agitation, rinsed with hot distilled water (100°C) until the pH became neutral (pH = 7), and soaked in acetone for 3 to 5 min. After soaking, the filter bags were spread out on a plate and placed under a ventilation hood for at least 30 min to evaporate the acetone before oven-drying the filter bags at 100°C for 8 h. Samples were cooled in desiccators for 20 min prior to weighing and recording the filter bag and sample residue. The weight obtained minus the initial bag weight constituted the alkaline hydrogen peroxide residue. The alkaline hydrogen peroxide residue was analyzed sequentially for acid-detergent fiber (ADF) and ADL according to the batch procedures outlined by ANKOM Technology Corp., Fairport, NY, USA. The ADL residue was ashed in a muffle furnace at 500°C for 8 h, and the mass of ash was subtracted from the mass of the ADL residue. The residue was then divided by the original sample weight to obtain ash-free APL. Samples were run in duplicate and when the coefficient of variation between replicates was greater than 5%, samples were rerun until the coefficient of variation was equal or less than 5%. In addition, samples were incubated 24 h instead of 48 h as it was suggested by [12], because the difference in alkaline hydrogen peroxide residue was not significantly different to justify the long incubation time based on preliminary samples we analyzed.

#### Procedure for ADIA

Approximately 0.5 ± 0.01 g of forage, ort, and fecal samples were placed into filter bags (ANKOM Corp. #F57) and analyzed for ADF according to the batch procedures outlined by ANKOM Technology Corp., Fairport, NY, USA. The ADF residue was then ashed in a muffle furnace at 500°C for 8 h. The ADIA concentrations were calculated as the residual ash divided by the initial sample weight.

### Marker recovery calculation, digestibility and fecal output estimation

The concentration of marker in consumed forage (M_fd,_ g/kg) was calculated using the following formula:

(1) $$\begin{array}{l} M_{\mathrm{fd}}\left(g/\mathrm{kg}\right)=[\left(M_{\mathrm{of}}\left(g/\mathrm{kg}\right)\times Q_{\mathrm{of}}\left(g\right)\right)-(M_{\mathrm{or}}\left(g/\mathrm{kg}\right)\\ \;\times Q_{\mathrm{or}}\left(g\right))]/\mathrm{DMI}\left(g\right) \end{array}$$

where M_of_ is the concentration of marker in hay offered; Q_of_ is the amount of hay offered; M_or_ is the concentration of marker in orts; Q_or_ is the amount of orts refused (Q_or_), and DMI is the actual DMI.

The recovery rates (R) of ADL, APL and ADIA, which are the ratios of the quantity of marker excreted in the feces per unit of marker consumed, were calculated using the following formula:

(2) $$\begin{array}{l} R\left(g/g\right)=\left(M_{\mathrm{fc}}\left(g/\mathrm{kg}\right)\times \mathrm{FO}\left(g\right)\right)/[(M_{\mathrm{of}}\left(g/\mathrm{kg}\right)\\ \;\times Q_{\mathrm{of}}\left(g\right))-\left(M_{\mathrm{or}}\left(g/\mathrm{kg}\right)\times Q_{\mathrm{or}}\left(g\right)\right)] \end{array}$$

where FO is the fecal DM excreted; M_fc_ is the marker concentration in feces.

The estimated DMD by internal marker was calculated using the following formula:

(3) $$\mathrm{DMD}\left(g/\mathrm{kg}\right)=1000\left(g/\mathrm{kg}\right)\times \left(1-M_{\mathrm{fd}}\left(g/\mathrm{kg}\right)/M_{\mathrm{fc}}\left(g/\mathrm{kg}\right)\right)$$

Estimates of FO were expressed as the ratio of the units of marker consumed per unit of marker excreted multiplied by the actual DMI according to the following formula:

(4) $$\mathrm{FO}\left(g\right)=\mathrm{DMI}\left(g\right)\times M_{\mathrm{fd}}\left(g/\mathrm{kg}\right)/M_{\mathrm{fc}}\left(g/\mathrm{kg}\right)$$

### Statistical analysis

Data for DMI, FO, and apparent DMD of the diet treatments and marker concentration in consumed hay and feces were analyzed as a 4 × 3 Youden Square design using PROC MIXED of SAS (SAS Inst. Inc., Cary, NC, USA, 2009) with diet as the main effect and period and block as random effects. The individual cow (n = 6 cows/ treatment) served as experimental unit for the diet effects and differences were considered significant at *P <* 0.05. Data of internal marker recovery (ADL, APL, and ADIA) and estimates of apparent DMD and FO were analyzed using the same experimental design with PROC MIXED, where diet, marker, and diet × marker interaction were included in the model. Block and period served as random effects. Results are reported as the least-squares means (LSMEANS). When significant differences were detected (*P <* 0.05), means were separated using the LSMEANS/PDIFF option in SAS (SAS Institute). The F-protected t-test was used to determine if the marker ratio estimates differed from 1.

## Results and discussions

### Chemical composition of bermudagrass hay diets

The nutrient composition of the bermudagrass hays fed to cattle during the experiment is presented in Table 1. Dry matter and hemicellulose concentrations were similar (*P* ≥ 0.19) across diet-treatments. Total ash and NDF appeared (*P =* 0.05 and 0.08, respectively) not to be impacted by diet. Concentration of organic matter was greater (*P* < 0.05) in L than in ML, MH, and H diets. Crude protein concentration was less (*P* < 0.05) on L diet, intermediate on ML and MH, and greater on H quality diets. Acid-detergent fiber and ADL appeared not to be related to diet quality as the greatest concentrations were obtained from L and H diets and the lowest concentrations from ML and MH diets. The higher fiber content on H was not expected as NDF and ADF are generally expected to be negatively correlated with CP concentration.

### Intake, digestibility, and fecal output

Data for DMI, FO, and apparent DMD for the bermudagrass hays with varying qualities are presented in Table 2. Forage DMI was affected by diet (*P =* 0.02), and DMI from MH and H was greater (*P* < 0.05) than from L. Apparent DMD tended (*P* = 0.08) to differ among diets while FO was not affected (*P =* 0.20) by diet.

**Table 2:** Dry matter intake (DMI), fecal output (FO), and DM digestibility (DMD) of bermudagrass hay with differing concentrations of crude protein (CP) fed to cattle based on total collection (n = 6)

**Item**	**Treatments**^**1**^				**SEM**^**2**^	***P*-value**
	**L**	**ML**	**MH**	**H**		
DMI, g/d	7,740^b^	9,020^ab^	10,210^a^	9,780^a^	762.0	0.02
FO, g/d	3,760	4,080	4,720	4,280	348.0	0.20
DMD, g/kg DM	504	550	538	564	15.4	0.08

^1^L, low CP hay (CP = 79 g/kg DM); ML, medium low CP hay (CP = 111 g/kg DM); MH, medium high CP hay (CP = 131 g/kg DM); and H, high CP hay (CP = 164 g/kg DM).

^2^SEM, standard error of the mean.

^ab^Means within a row without a common superscript letter differ at *P* < 0.05.

Differences in DMI were unexpected, because hay was offered at restricted intake (20 g/kg of body weight). However, the low-quality forage may have reduced the rate of passage which then reduced DMI. The DMI response may have affected the rate of passage (k_p_), which in turn mitigated the expected difference in DMD [16]. Also, the NDF concentrations of the four dietary treatments were not consistent with CP concentrations.

### Internal marker concentration in hay and feces

The concentrations of ADL, APL, and ADIA in the different bermudagrass hays and in the feces are presented in Table 3. Concentrations of ADL were greater (*P* < 0.05) for L and H diets compared with ML and MH diets. Fecal ADL concentrations appeared not to be related (*P* = 0.06) to diet quality. Concentrations of APL in hay and feces were similar (*P* ≥ 0.16) by diet. Conversely, diet affected (*P* < 0.01) ADIA concentration in hay consumed and in feces. Fecal ADIA concentrations did not appear to be related to forage CP concentrations, as the greatest (*P* < 0.05) fecal concentrations of ADIA resulted from cows offered the ML and MH hays.

**Table 3:** Concentration (g/kg dry matter, DM) of internal markers in consumed bermudagrass hays of varying crude protein (CP) concentrations and associated feces (n = 6)

**Item**^**2**^	**Treatments**^**1**^				**SEM**^**3**^	***P*-value**
	**L**	**ML**	**MH**	**H**		
ADL_fd_	43^a^	32^b^	32^b^	38^a^	1.9	0.01
ADL_fc_	93	85	87	94	2.6	0.06
APL_fd_	26	24	22	25	1.2	0.16
APL_fc_	59	53	52	60	4.6	0.17
ADIA_fd_	25^b^	32^a^	27^b^	20^c^	2.8	0.01
ADIA_fc_	51^b^	65^a^	60^a^	54^b^	4.2	0.01

^1^L, low CP hay (CP = 79 g/kg DM); ML, medium low CP hay (CP = 111 g/kg DM); MH, medium high CP hay (CP = 131 g/kg DM); and H, high CP hay (CP = 164 g/kg DM).

^2^ADL_fd_, acid-detergent lignin in the forage; ADL_fc_, acid-detergent lignin in feces; APL_fd_, alkaline-peroxide lignin in the forage; APL_fc_, alkaline-peroxide lignin in feces; ADIA_fd_, acid-detergent insoluble ash in the forage; ADIA_fc_, acid-detergent insoluble ash in feces.

^3^SEM, standard error of the mean.

^abc^Means within a row without a common superscript letter differ at *P <* 0.05.

The ADL concentrations obtained from the hays used in this study varied between 32 and 43 g/kg DM, and were similar to those reported by other authors [17]. The average APL concentrations in hay and feces in this study were 24 and 56 g/kg DM, respectively. A similar fecal APL concentration (49 g/kg) was reported [9] for cows fed prairie hay. Slightly lower APL concentrations in forage and feces were obtained (18 and 46 g/kg) from smooth bromegrass (*Bromus inermis* Leyss.) and 19 and 45 g/kg from prairie hay [12], respectively. Greater APL concentrations (39 g/kg) in forage and similar values (55 g/kg) in feces were obtained [8] from immature and dormant grass when forage samples were incubated in alkaline hydrogen peroxide solution before acid-detergent extraction. As expected in this study, concentrations of APL were smaller than ADL due to the removal of core and non-core lignin fractions [18] when forage samples were incubated in alkaline hydrogen peroxide before ADF extraction. It has been estimated that up to half of the lignin in roughage may be removed with alkaline hydrogen peroxide treatment [19,20].

Average concentrations of ADIA in feed and feces for this study were 26 and 58 g/kg of DM, respectively. Fecal ADIA concentrations of 59, 58, 52, and 46 g/kg DM were reported from lambs fed alfalfa (*Medicago sativa* L.) [21], and prairie hay [22], steers fed tall grass prairie hay [23], and cattle fed alfalfa [22], respectively. Lower concentrations of ADIA in feed and feces (3.8 and 13.2 g/kg DM) were observed from cattle fed various dairy cattle diets [24].

### Recovery of internal markers

The diet × marker interaction only tended (*P* = 0.06) to affect marker recovery (Table 4); therefore, only main effects will be discussed. Recovery of ADL was greater (*P <* 0.05) than that of ADIA and APL, but recovery of ADIA and APL did not differ (*P* = 0.42) from each other. Dietary treatments did not alter (*P* = 0.47) the recovery of ADL, APL, and ADIA. Furthermore, the mean recovery for ADL differed (*P* < 0.01) from 1, that of APL tended (*P* = 0.07) to differ, whereas ADIA recovery did not differ from 1 (*P* = 0.20).

**Table 4:** Recovery (g/g) of internal markers in feces for each bermudagrass hay treatment (n = 6)

**Item**^**2**^	**Treatments**^**1**^						***P*-value**^**5**^			
	**L**	**ML**	**MH**	**H**	**Mean**^**3**^	**SEM**^**4**^	**D**	**M**	**D × M**	***P*** **(≠1)**^**6**^
ADL	1.10	1.20	1.29	1.05	1.16^a^	0.035	0.47	<0.01	0.06	<0.01
APL	1.10	1.02	1.07	1.05	1.06^b^					0.07
ADIA	1.01	0.96	1.03	1.12	1.03^b^					0.20

^1^L, low crude protein hay (CP = 79 g/kg DM); ML, medium low CP hay (CP = 111 g/kg DM); MH, medium high CP hay (CP = 131 g/kg DM); and H, high CP hay (CP = 164 g/kg DM).

^2^ADL, acid-detergent lignin; APL, alkaline-peroxide lignin; ADIA, acid-detergent insoluble ash.

^3^Average per markers across different treatments.

^4^SEM, standard error of the mean.

^5^D, diet effect; M, marker effect; and D × M, diet by marker interaction.

^6^Probability of the t-test with the null hypothesis that mean recovery is different from 1 g/g (α = 0.05).

^ab^Means within a column without a common superscript letter differ at *P <* 0.05.

In a previous study [1], steers fed alfalfa cubes had incomplete ADL recovery (0.52), whereas steers consuming tall wheatgrass [*Agropyron elongatum* (Host) Beauv.] plus soybean [*Glycine max* (L.) Merr.] meal had a positive ADL recovery (1.16). Fecal recoveries of ADL were 0.92, 1.07, and 1.15 in steers fed alfalfa, bromegrass, and prairie hay, respectively [12]. Incomplete ADL fecal recoveries (0.78 and 0.94) were obtained from lambs fed prairie hay and alfalfa hay, respectively [25]. However, ADL fecal recovery close to 1 was reported [26] for ryegrass (*Lolium multiflorum* Lam.) diets. The positive recovery of ADL may be attributable to the formation of an artifact during the transit of ingested forage in the gastrointestinal tract of ruminants [27,28]. Nearly 50% of the lignin in forage may conjugate with carbohydrates and form an insoluble complex that will be measured in feces as lignin [29]. The incomplete ADL recovery may be attributable to the biodegradation of lignin during its transit in the gastrointestinal tract [7,30] and the formation of a soluble lignin-carbohydrate complex in the rumen environment [31,32]. Also, incomplete fecal recovery of lignin as an internal marker may be associated with its low concentration in immature forages and the variability in lignin content in different plant parts.

Fecal recovery of APL averaged 1.06 in the present study. In other studies, a recovery of 0.98 with a range from 0.82 to 1.18 was reported using lambs that were fed prairie hay [9], and a recovery of 0.98 was achieved [8] using steers fed dormant big bluestem grass (*Andropogon gerardii* Vitman), following the addition of alkaline hydrogen peroxide before acid-detergent extraction. This procedure improved the recovery of lignin from plants and feces. In a subsequent study [12], APL recovery rates were 1.06 and 0.93 from steers fed smooth bromegrass, and prairie hay, respectively. An APL fecal recovery of 1 was achieved in sheep fed ad libitum tall fescue [*Lolium arundinaceum* (Schreb.) Darbysh] hay, although actual and predicted digestibility values were different [33]. However, incomplete APL fecal recovery (0.79) was observed from cows fed finger millet (*Eleusine coracona*) straw with supplements [34].

In our study, the ADIA fecal recovery averaged 1.03. Similar fecal recovery of ADIA (0.99) was reported [35] for steers fed alfalfa, bermudagrass and prairie hay without supplements. Also, ADIA fecal recovery rate was close to 1 (1.05 ± 0.025) for lambs fed alfalfa [21] and steers fed forage-based diets with different levels of supplementation [22]. Supplementation did not have an effect on ADIA recovery [22]. However, ADIA recovery of 0.94 was reported in cattle consuming supplemented finger millet straw [34]. Although over-recovery may occur owing to soil contamination, ADIA had the potential to perform as an internal marker due to rapid analysis, low cost, and low analytical error compared to lignin based-markers [10].

### Estimates of FO and apparent DMD

Estimates of FO differed by marker (*P =* 0.01; Table 5) and diet (*P =* 0.01), but there was no diet × marker interaction (*P =* 0.50).Therefore, only main effect will be discussed. Fecal output estimates by APL and ADIA were not different from each other (*P =* 0.74) and not different (*P* ≥ 0.39) from observed FO, while ADL estimates differed (*P* < 0.05) from total fecal collection and underestimated FO. Estimates of DMD were affected by marker (*P =* 0.01) and diet (*P =* 0.01), but not the diet × marker interaction (*P* = 0.22), indicating that ranges in forage CP did not affect the ability of the markers to predict DMD. The DMD estimates of ADIA and APL were not different (*P =* 0.54) from each other and not different (*P* ≥ 0.28) from observed DMD, whereas ADL overestimated (*P* < 0.05) DMD. In general, estimates of ADL were different from all other estimates and overestimated the apparent DMD by over 10% while underestimating FO by 13%.

**Table 5:** Estimates of fecal output (FO, g/d) and dry matter digestibility (DMD, g/kg) using different internal markers compared with values derived from total collection (TC) (n = 6)

**Item**^**2**^	**Treatments**^**1**^						***P*-value**^**4**^		
	**L**	**ML**	**MH**	**H**	**Average**	**SEM**^**3**^	**D**	**M**	**D × M**
Fecal output									
TC	3,760	4,080	4,720	4,280	4,210^a^	122.0	0.01	0.01	0.50
ADL	3,370	3,470	3,740	4,040	3,660^b^				
APL	3,510	4,090	4,590	4,050	4,060^a^				
ADIA	3,710	4,290	4,660	3,810	4,170^a^				
Average	3,588^e^	3,983^d^	4,428^c^	4,045^d^					
Dry matter digestibility									
TC	504	550	538	564	539^b^	11.1	0.01	0.01	0.22
ADL	547	613	635	592	597^a^				
APL	543	539	552	590	556^b^				
ADIA	507	520	543	617	547^b^				
Average	525^e^	555^de^	567^cd^	591^c^					

^1^L, low CP hay (CP = 79 g/kg DM); ML, medium low CP hay (CP = 111 g/kg DM); MH, medium high CP hay (CP = 131 g/kg DM); and H, high CP hay (CP = 164 g/kg DM).

^2^ADL, acid-detergent lignin; APL, alkaline-peroxide lignin; ADIA, acid-detergent insoluble ash.

^3^SEM, standard error of the mean.

^4^D, diet effect; M, marker effect; D × M, diet by marker interaction.

^ab^Means within a column without a common superscript letter differ at *P <* 0.05.

^cde^Means within a row without a common superscript letter differ at *P <* 0.05.

Based on this information, ADIA and APL are potential internal markers that can predict FO and DMD of bermudagrass hay having a wide range of CP concentrations, while ADL does not appear to be useful for predicting either FO or DMD of bermudagrass hay. Generally, the ability of an internal marker to estimate FO and DMD reflects its fecal recovery. Inability for lignin (ADL) to accurately predict DMD was reported by some authors [1,8,35]. In this study, APL produced estimates of FO and DMD similar to those of total fecal collection. Other authors reported similar results [8,9,12], whereas DMD was underestimated by APL in one study because of incomplete fecal recovery [34]. Acid-detergent insoluble ash was the best in predicting FO and DMD. This marker also accurately predicted the DMD of various diets fed to lambs, dairy cattle, and steers [21,24,36]. According to the results of this study, ADIA and APL can be used to assess the digestibility and fecal output in cattle fed bermudagrass hays regardless of hay quality. Further studies may evaluate the potential of these markers on different forage species, levels of feeding or supplementation.

## Conclusion

Acid-detergent insoluble ash and APL appear to be appropriate internal markers for predicting fecal output and dry matter digestibility by cattle fed bermudagrass hay of varying quality. Having such internal markers will facilitate larger studies involving greater numbers of animals and forages to determine the digestibility by applying the marker ratio technique. These studies can then be used to develop more accurate equations to predict energy values of forages based on the relationship of dietary components to digestibility across a wide range of forages.

## Abbreviations

ADIA: Acid-detergent insoluble ash; APL: Alkaline-peroxide lignin; ADL: Acid-detergent lignin; FO: Fecal output; DMD: dry matter digestibility; CP: Crude protein; ADF: Acid-detergent fiber; L: Low crude protein; ML: Medium low crude protein; MH: Medium high crude protein; H: High protein; DMI: Dry matter intake.

## Competing interests

The authors declare that they have no competing interests.

## Authors’ contributions

JK conceived the study, carried out the experimental trial, performed the statistical analysis, and drafted the initial manuscript. DP and KPC, conceived and participated in design of the study, carried out experimental trial, and statistical analysis and helped to draft the manuscript. EBK, CPW, SG, and JJ: conceived and participated in design of the study and revised the manuscripts; ANY and RR carried out the experimental trial. All authors read and approved the final manuscript.
